# CHMP5 attenuates osteoarthritis via inhibiting chondrocyte apoptosis and extracellular matrix degradation: involvement of NF-κB pathway

**DOI:** 10.1186/s10020-024-00819-6

**Published:** 2024-04-25

**Authors:** Weilu Gao, Rui Liu, Keke Huang, Wenhan Fu, Anquan Wang, Gongwen Du, Hao Tang, Li Yin, Zongsheng S. Yin

**Affiliations:** 1 Department of Orthopedics, The First Affiliated Hospital of Anhui Medical University, No. 218, Jixi Road, Hefei, Anhui China; 2 Department of Orthopedics, Wan Bei General Hospital of Wanbei Coal power Group, Suzhou, Anhui China

**Keywords:** Osteoarthritis, Charged multivesicular body protein 5, Extracellular matrix degradation, Chondrocyte apoptosis, Nuclear factor kappa-B signaling pathway

## Abstract

**Background:**

Osteoarthritis (OA), the most common joint disease, is linked with chondrocyte apoptosis and extracellular matrix (ECM) degradation. Charged multivesicular body protein 5 (CHMP5), a member of the multivesicular body, has been reported to serve as an anti-apoptotic protein to participate in leukemia development. However, the effects of CHMP5 on apoptosis and ECM degradation in OA remain unclear.

**Methods:**

In this study, quantitative proteomics was performed to analyze differential proteins between normal and OA patient articular cartilages. The OA mouse model was constructed by the destabilization of the medial meniscus (DMM). In vitro, interleukin-1 beta (IL-1β) was used to induce OA in human chondrocytes. CHMP5 overexpression and silencing vectors were created using an adenovirus system. The effects of CHMP5 on IL-1β-induced chondrocyte apoptosis were investigated by CCK-8, flow cytometry, and western blot. The effects on ECM degradation were examined by western blot and immunofluorescence. The potential mechanism was explored by western blot and Co-IP assays.

**Results:**

Downregulated CHMP5 was identified by proteomics in OA patient cartilages, which was verified in human and mouse articular cartilages. CHMP5 overexpression repressed cell apoptosis and ECM degradation in OA chondrocytes. However, silencing CHMP5 exacerbated OA chondrocyte apoptosis and ECM degradation. Furthermore, we found that the protective effect of CHMP5 against OA was involved in nuclear factor kappa B (NF-κB) signaling pathway.

**Conclusions:**

This study demonstrated that CHMP5 repressed IL-1β-induced chondrocyte apoptosis and ECM degradation and blocked NF-κB activation. It was shown that CHMP5 might be a novel potential therapeutic target for OA in the future.

**Supplementary Information:**

The online version contains supplementary material available at 10.1186/s10020-024-00819-6.

## Introduction

Osteoarthritis (OA) is the most prevalent chronic arthritis characterized by joint pain, stiffness, and dysfunction (Emery et al. 2019; Hunter and Bierma-Zeinstra 2019). OA is a multifactorial disease caused by genetics, joint trauma, aging, and obesity (Abramoff and Caldera 2020; Theeuwes et al. 2021). OA severity is characterized by extracellular matrix (ECM) decomposition, chondrocyte apoptosis, synovitis, and subchondral bone pathology (Dilley et al. 2023; Jiang et al. 2020). It exerts a heavy burden on society and individuals (Hunter et al. 2014; Martel-Pelletier et al. 2016). The treatments for OA center on pain amelioration and joint replacement (Cao et al. 2020; Queen 2017), which is not satisfactory due to the lack of effective disease modifying approaches (Katz et al. 2021). It is urgently needed to discover novel therapeutic strategies for OA.

The chondrocytes are capable of synthesizing ECM such as proteoglycans and collagen, which are the major articular cartilage components (He et al. 2020; Li et al. 2017). However, interleukin-1 beta (IL-1β), a pro-inflammatory cytokine, has been reported to destruct articular cartilages by constricting the synthesis of anabolic substances like collagen and proteoglycans, augmenting the production of catabolic substances such as a disintegrin and metalloprotease with thrombospondin motifs (ADAMTS) and matrix metalloproteinases (MMPs), as well as promoting chondrocyte apoptosis (Goldring et al. 1994; Kapoor et al. 2011; Mengshol et al. 2000; Mirza et al. 2018). Furthermore, previous studies have demonstrated that nuclear factor kappa-B (NF-κB) signaling pathway is a potential target for OA treatment (Lepetsos et al. 2019). For instance, stimulator of interferon genes knockdown decelerates IL-1β-triggered senescence, apoptosis, and ECM degradation in OA via suppressing NF-κB signaling axis (Guo et al. 2021). Knockdown of Forkhead box M1 represses the IL-1β-induced inflammatory response in human osteoarthritis chondrocytes, and the underlying mechanism is involved in the inhibition of NF-κB activation (Zeng et al. 2019). Silencing LIM homeobox transcription factor 1 beta suppresses cell apoptosis and inflammatory response in IL-1β-induced human OA chondrocytes through NF-κB pathway (Mu et al. 2022). Chondroitin sulfate, a component of cartilage, is shown to have anti-inflammatory action on chondrocytes and to affect NF-κB pathway (da Cunha et al. 2017). These findings indicate that targeting the inhibition of IL-1β-induced ECM degradation, chondrocyte apoptosis and NF-κB pathway contribute to treat OA. However, several IL-1β antagonists have not achieved ideal results in clinical trials. For example, lutikizumab, a novel immunoglobulin agent that targets and inhibits IL-1α and IL-1β, improves pain only slightly and fails to slow cartilage loss or reduce synovitis (Fleischmann et al. 2019; Kloppenburg et al. 2019). Exploring new active molecules targeting IL-1β inhibition becomes an attractive strategy for combating OA.

In the present study, we identified the differentially expressed proteins via proteomic analysis and discovered downregulated charged multivesicular body protein 5 (CHMP5) in the articular cartilages of OA patients (log_2_ fold change| > 0.8, *p* < 0.05). CHMP5, also known as peptide nucleic acids 2 (PNAS-2), belongs to the chromatin-modifying protein/charged multivesicular body protein (CHMP) family (Wang et al. 2006) and is in charge of the final conversion of late endosomal endocytic multivesicular bodies to lysosomes (Shim et al. 2006). Silencing CHMP5 elevates the content of caspase-8 and caspase-9 in acute myeloid leukemia cells, indicating that CHMP5 is able to prevent cell apoptosis (Shahmoradgoli et al. 2011). Consistently, CHMP5 functions as an anti-apoptotic gene in leukemia (Wang et al. 2006). Multiple studies have demonstrated that NF-κB signaling pathway is modulated by CHMP5 (Shim et al. 2006; Wang et al. 2013). CHMP5 deficiency activates NF-κB pathway in myelomonocytic leukemia cells (Wang et al. 2013). NF-κB activation induced by the inflammatory cytokines tumor necrosis factor alpha (TNFα) and IL-1β is inhibited by CHMP5 overexpression during mouse embryogenesis (Hayden and Ghosh 2004; Shim et al. 2006). In view of the previous studies, we hypothesized that CHMP5 played an essential role in OA progression.

Herein, we aimed to investigate the effects of CHMP5 on chondrocyte apoptosis and ECM metabolism, as well as the potential molecular mechanisms in OA. Our study might provide a novel therapeutic target for OA treatment.

## Materials and methods

### Human cartilage and chondrocyte culture

The normal human articular cartilages were obtained from the femoral heads of 27 patients with femoral neck fractures undergoing total hip replacement or artificial femoral head replacement surgery, but with no significant clinical and imaging features of OA (18 women, 9 men; 53–88 years old; mean 73.4 years; Kellgren-Lawrence grade, 0). The OA human articular cartilages were obtained from 30 patients undergoing total knee arthroplasty (22 women, 8 men; 49–81 years old; mean 66.2 years; Kellgren-Lawrence grade, III or IV). The cartilages were cut into approximately 1 mm^3^ pieces, and then they were digested by 0.25% trypsin (Beyotime, Shanghai, China) for 30 min. Next, the samples were incubated with 0.2% collagenase II (Sigma, St. Louis, MO, USA) at 37 °C overnight. The obtained chondrocytes were cultured in the Dulbecco’s Modified Eagle Medium/Nutrient Mixture F-12 (DMEM/F12; Biosharp, Hefei, China) containing 15% fetal bovine serum (FBS; Tianhang, Huzhou, China) in 5% CO_2_ at 37℃. Finally, the chondrocytes at first passage were selected for the further experiments. Informed consents were obtained from the donors. Our study was conducted based on Declaration of Helsinki. Ethical approval was obtained from Clinical Medical Research Ethics Committee of the First Affiliated Hospital of Anhui Medical University (PJ2019-06-06).

### Cell adenovirus infection

Recombinant adenovirus vectors expressing CHMP5 (Ad-CHMP5) were constructed by General Biosystems (Chuzhou, China). Briefly, DNA fragments encoding CHMP5 (NM_016410) were subcloned into the pShuttle-CMV vector (Fenghui, Changsha, China), and recombined with pAdEasy-1 in BJ5183-AD-1 cells (Huayueyang, Beijing, China). The linearized vectors carrying CHMP5 encoding fragments were then transfected into HEK-293A cells (iCell, Shanghai, China) via Lipofectamine 3000 (Invitrogen, USA) to produce adenoviral particles. The empty adenoviral vector (Ad-NC) was used as a negative control. Adenoviruses containing short hairpin RNA (shRNA) targeting CHMP5 (Ad-shCHMP5) or none-targeting shRNA (Ad-shNC) were also generated by General Biosystems (Chuzhou, China). The sequences were as follows: Ad-shCHMP5: 5’-GGATGAAGATGATTTAGAAGC-3’; Ad-shNC: 5’-TTCTCCGAACGTGTCACGT-3’. The titers reached 1.6 × 10^9^ pfu/mL for Ad-CHMP5, 2.8 × 10^9^ pfu/mL for Ad-NC, 2 × 10^9^ pfu/mL for Ad-shCHMP5 and 2.2 × 10^9^ pfu/mL for Ad-shNC, respectively. Chondrocytes were infected with adenoviruses at 100 multiplicity of infection (MOI).

To mimic inflammatory stimulation, chondrocytes were exposed to IL-1β (10 ng/mL; Sinobiological, Beijing, China) for 6 h, 12 h, 24–48 h. To inhibit protein degradation mediated by ubiquitin-proteasome system, chondrocytes were treated with MG132 (10 µM; Aladdin, Shanghai, China) for 4 h.

### Experimental mice

OA was induced in the male C57BL/6 mice (10–12 weeks) using destabilization of the medial meniscus (DMM) (Glasson et al. 2007). The mice were anesthetized by isoflurane inhalation (3% for induction and 1.5% for maintenance). The right knee joints of the mice were exposed by incision from the medial side of the patella, and the medial meniscotibial ligaments were transected by micro-surgical scissors. In sham surgery, the ligaments were visualized without further damage. Finally, the incisions were sutured. All mice were euthanized at 4 and 8 weeks after the surgery, and all knee joints were collected to detect CHMP5 expression. For the effect of CHMP5 on OA mice, starting at 10 days post-operation, Ad-CHMP5 was injected into the mouse articular cartilages once a week for the next three weeks. The Ad-NC injection was used as a control. The mice were sacrificed 8 weeks after the operation. The knee joints were isolated and gathered for further histological analysis. Meloxicam (5 mg/kg) was administered subcutaneously about 1 h before the DMM operation and once a day for 3 days after surgery. Ethical approval was obtained from the Experimental Animal Ethics Committee of Anhui Medical University.

### Proteomic analysis

The articular cartilages of 3 OA patients (Kellgren-Lawrence grade, III or IV) and 3 normal cartilages (Kellgren-Lawrence grade, 0) were grinded with liquid nitrogen, and then weighed 150 mg. The tissues were lysed using SDT lysis buffer. The supernatant was collected after centrifuging (20,000 g, 4℃). The samples were digested in trypsin and marked using TMT 6plex kit (Thermo Fisher Scientific, Shanghai, China). Then, labeled peptides were fractionated by the Pierce™ High pH Reversed-Phase Peptide Fractionation Kit (Thermo Fisher Scientific, Shanghai, China) for liquid chromatography-tandem mass spectrometry (LC-MS/MS) analysis. The Easy nLC 1200 chromatographic system was applied to separate the peptides. Data-dependent acquisition (DDA) analysis was performed by a Q-Exactive HF-X mass spectrometer (Thermo Fisher Scientific, Shanghai, China). Proteome Discoverer 2.4 software was used to retrieve the data and quantify the protein. The false discovery rate (FDR) was set to 1% for protein and peptide spectrum matches.|log_2_ fold change| > 0.8 and *p* < 0.05 were considered as the criterions for screening differential proteins.

### Histological analysis

The mouse cartilage tissues were decalcified in 10% disodium ethylene diamine tetraacetic acid (Biosharp, Hefei, China), embedded in paraffin and cut into 5-µm-thick sections. All tissues were stained with safranin O-fast green. The articular cartilage destruction was evaluated using the Osteoarthritis Research Society International (OARSI) scoring system by estimating the highest observed scores based on a previous study (Glasson et al. 2010). For immunohistochemistry (IHC), the sections embedded in paraffin were dewaxed, hydrated, and inoculated with 3% hydrogen peroxide. Thereafter, the sections were blocked with 1% bovine serum albumin (Sangon, Shanghai, China) for 15 min at room temperature and incubated with the primary antibodies against CHMP5 (1:50), collagen II (1:50) and matrix metallopeptidase 13 (MMP13; 1:50) purchased from Affinity Biosciences (Changzhou, China) at 4 °C overnight. Then, the slides were inoculated with horseradish peroxidase (HRP)-labeled goat anti-rabbit IgG (1:500; Thermo Fisher Scientific, Shanghai, China) at 37 °C for 1 h. Next, the 3, 3-diaminobenzidine tetrahydrochloride (Maixin, Fuzhou, China) was used as a chromogenic agent. The images were captured with a microscope (Olympus, Japan).

### Real time PCR

Total RNA was extracted from the mouse articular cartilages and the human articular chondrocytes using the TRIpure reagent (BioTeke, Beijing, China). The corresponding cDNA was obtained using BeyoRT II M-MLV Reverse Transcriptase (Beyotime, Shanghai, China). The primer sequences are as follows: mouse-CHMP5 forward primer: 5’-CATTGGGACGGTGGATA-3’, mouse-CHMP5 reverse primer: 5’-GGTTGTCTCGCTGTTGC-3’; human-CHMP5 forward primer: 5’-GGCACGGTGGACAGTAG-3’, human-CHMP5 reverse primer: 5’-ACTCGCAAGGCTTTCTG-3’. Glyceraldehyde 3-phosphate dehydrogenases (GAPDHs) were used as the endogenous controls. The relative expression levels were calculated by the 2^−ΔΔCt^ method.

### Cell counting kit 8 (CCK-8)

The chondrocytes were seeded in 96-well plates (4 × 10^3^ cells per well) and cultured overnight. After that, IL-1β (10 ng/mL) was applied to the transfected cells for 24 h in 5% CO_2_ at 37℃. CCK8 reagent (10 µL; Solarbio, Beijing, China) was added into each well. Subsequently, the samples were incubated for 2 h. The OD values were determined at 450 nm by a microplate reader (BioTek, USA).

### Flow cytometric

Annexin V-FITC and phycoerythrin (PE) were used to stain the chondrocytes referring to the cell apoptosis detection kit (KeyGene, Nanjing, China). The cells were analyzed through a NovoCyte Flow Cytometer (Aceabio, USA).

### Immunofluorescence (IF) analysis

The chondrocytes were fixed with 4% paraformaldehyde (Sinopharm, Shanghai, China) for 15 min, permeabilized with 0.1% Triton X-100 (Beyotime, Shanghai, China) for 30 min, and blocked by 1% bovine serum albumin (Sangon, Shanghai, China) for 15 min. The samples were incubated with collagen II (1:100; Affinity, Changzhou, China) at 4 °C overnight, followed by incubation with Cy3-conjugated goat anti-rabbit IgG (1:200; Invitrogen, USA) for 1 h. Images were taken by a fluorescence microscope (Olympus, Japan).

### Co-immunoprecipitation (Co-IP)

The chondrocytes were lysed with lysis buffer (Beyotime, Shanghai, China) on ice for 5 min. The cell lysates were centrifuged at 10,000 g for 5 min. The collected supernatants were incubated with rabbit anti-CHMP5 (1:1000, Affinity, Changzhou, China), anti-NF-κB inhibitor alpha (IκBα1; 1:1000, ABclonal, Wuhan, China), anti-ubiquitin specific peptidase 15 (USP15; 1:500, ABclonal, Wuhan, China) or anti-Ubi (1:1000, ABclonal, Wuhan, China) antibodies at 4 °C overnight, after which they were incubated with protein A agarose beads. Subsequently, western blot was performed as follows.

### Western blot

Total articular cartilages and chondrocytes were lysed in radioimmunoprecipitation assay (RIPA) buffer containing 1 mM phenylmethanesulfonyl fluoride (PMSF). For the examination of NF-κB p65, the cytoplasm and nucleus were separated with cytoplasmic and nuclear extraction reagents according to the Nuclear Protein Extraction Kit (Solarbio, Beijing, China). The proteins were separated by sodium dodecyl sulphate polyacrylamide gel electrophoresis (SDS-PAGE) and transferred onto polyvinylidene difluoride (PVDF) membranes (Millipore, USA) afterwards. RIPA, PMSF and SDS-PAGE were purchased from Solarbio Science & Technology (Beijing, China). The membranes were blocked by 5% (M/V) skimmed milk and then incubated with the anti-rabbit primary antibody CHMP5 (1:1000), collagen-II (1:500), cleaved poly (ADP-ribose) polymerases (PARP; 1:500), cleaved caspase-3 (1:1000), aggrecan (1:400) from Affinity Biosciences (Changzhou, China), SOX9 (1:1000), IκBα (1:1000), p-p65 (1:1000), p65 (1:500), MMP13 (1:1000), matrix metallopeptidase 3 (MMP3; 1:2000), ADAMTS5 (1:500) from ABclonal (Wuhan, China), Histone H3 (1:5000) from Gene Tex (USA) as well as anti-mouse control GAPDH (1:10000) from proteintech (Wuhan, China) overnight at 4 °C. The secondary antibody HRP-conjugated goat anti-rabbit IgG (1:3000) and anti-mouse IgG (1:3000) from Solarbio Science & Technology (Beijing, China) were incubated with the membranes at 37 °C for 1 h. The results were imaged using the enhanced chemiluminescence luminous (ECL; Solarbio, Beijing, China).

### Statistical analysis

Statistical analysis was performed with GraphPad Prism software, and the data were expressed as mean ± SD. Differences between two groups were evaluated by unpaired t test. Differences among multiple groups were assessed using one-way ANOVA with Tukey’s test. The non-parametric Mann-Whitney test was used to compare the OARSI scores and IHC staining scores between two groups. *P* < 0.05 was regarded as statistical significance.

## Results

### Identification and function analysis of the differential proteins in OA articular cartilages

**Fig. 1:**
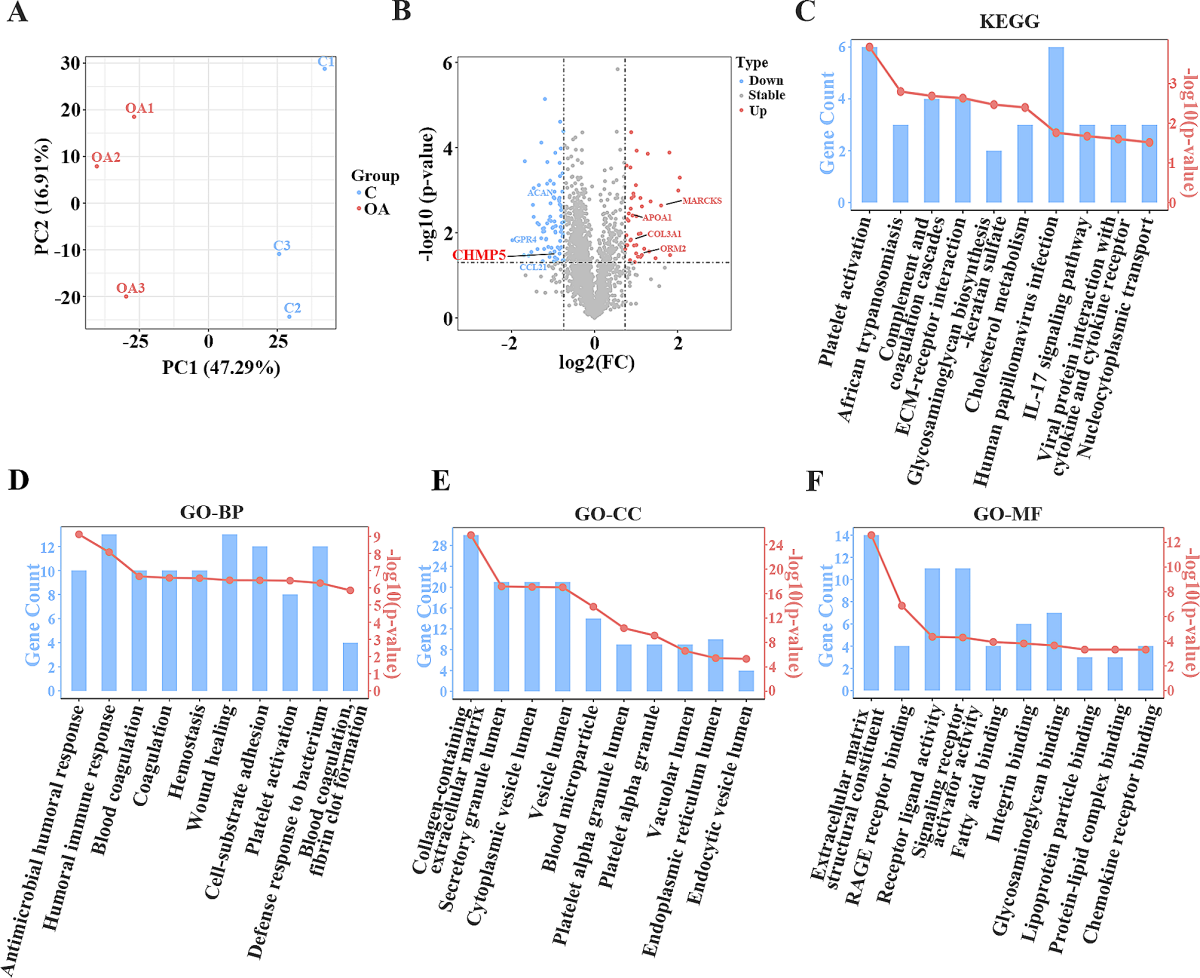
Proteomic analysis of the articular cartilage tissues in OA patients. (**A**) A 2D plot of PCA in control and OA groups. (**B**) Volcano plots of the differential proteins. (**C**) KEGG enrichment analysis. (**D**) Biological process analysis (GO-BP). (**E**) Cellular component analysis (GO-CC). (**F**) Molecular function analysis (GO-MF)

As shown in Fig. S1A and B, the correlation of samples was analyzed. A matrix of scatter plots and Pearson correlation coefficient of protein intensities of the control and OA groups (three parallel samples for each group) showed high repeatability of the samples (Pearson correlation coefficient *R* > 0.97; Fig. S1A). A histogram of intensity for each sample in TMTTM-based proteomic analysis was shown in Fig. S1B. Heat map analysis was performed based on the proteomic data (Fig. S1C). The sample distribution was also presented in the principal component analysis (PCA) plot (Fig. 1A). We identified 151 proteins that were differentially expressed in the OA group compared with the control group, including 86 up-regulated and 65 down-regulated proteins, respectively (|log_2_ fold change| > 0.8, *p* < 0.05). The results indicated that CHMP5 was down-regulated in OA patients. The volcano plot was presented in Fig. 1B. Kyoto Encyclopedia of Genes and Genomes (KEGG) enrichment analysis showed that platelet activation, human papillomavirus infection, complement and coagulation cascades and ECM-receptor interaction were the most enriched pathways (Fig. 1C). The differential proteins were mainly classified in the terms of biological processes (BP), cellular components (CC) and molecular functions (MF) in the Gene Ontology (GO) enrichment analysis (Fig. 1D-F), in which humoral immune response, wound healing and cell-substrate adhesion, in the terms of BC; collagen-containing extracellular matrix, secretory granule lumen and cytoplasmic vesicle lumen in the terms of CC; extracellular matrix structural constituent, receptor ligand activity and signaling receptor activator activity, in the terms of MF, were enriched in OA patients (Fig. 1D-F).

### CHMP5 is reduced in the articular cartilages of humans and mice with OA

**Fig. 2:**
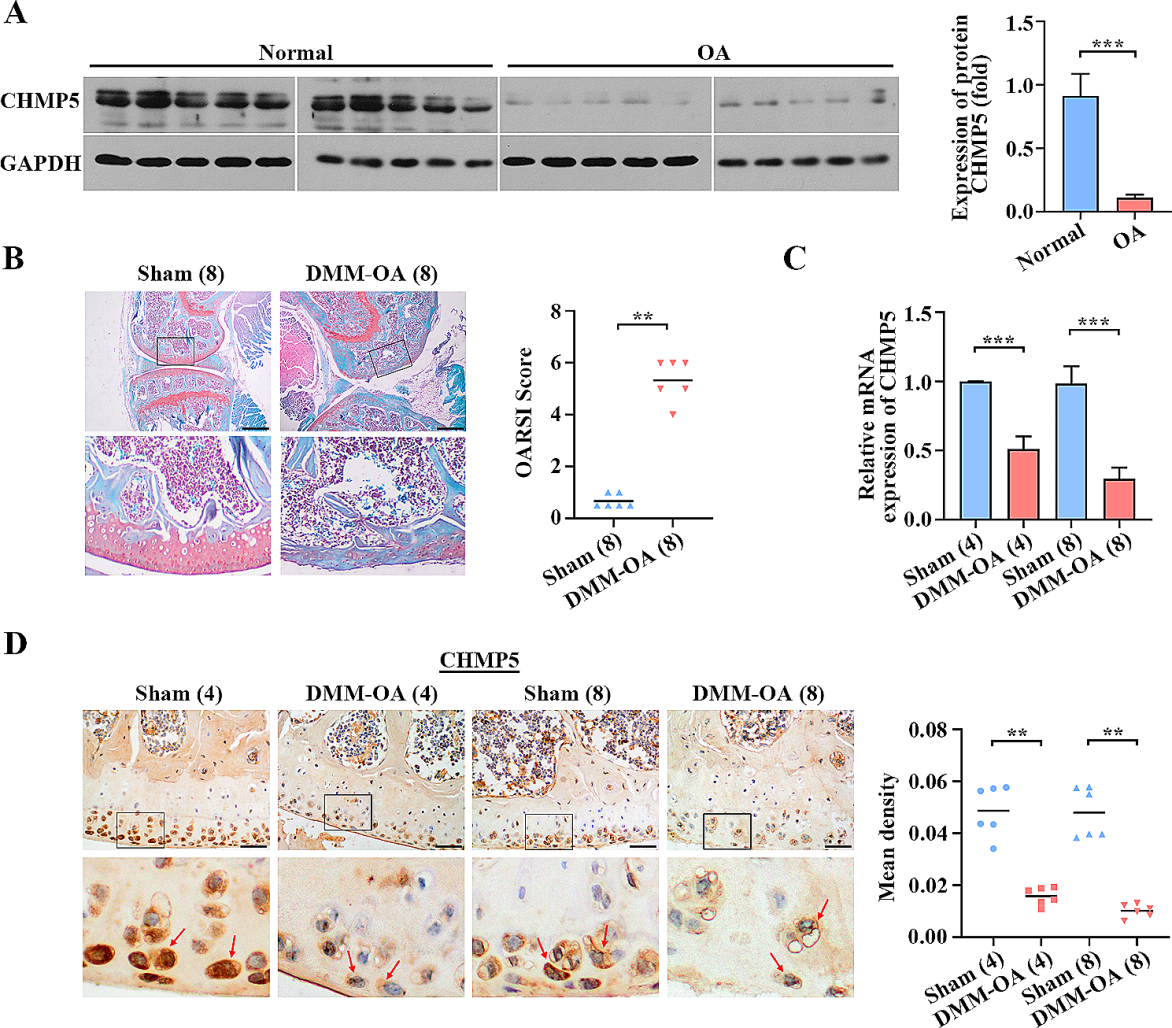
The expression of CHMP5 was decreased in OA human and mouse articular cartilages. (**A**) The protein content of CHMP5 was determined in normal and OA patient cartilages by western blot. (**B**) Safranin O-fast green staining images and OARSI score of articular cartilages from the sham and DMM mice at 8 weeks after surgery (Mann-Whitney test). Bar: 500 μm. (**C**) Real time PCR and (**D**) immunohistochemistry (IHC) showed the expression of CHMP5 in the mouse cartilages (Mann-Whitney test). Bar: 50 μm. **P < 0.01. ***P < 0.001

CHMP5 proteins were decreased in the articular cartilages derived from OA patients compared to the healthy tissues (*P* < 0.001, Fig. 2A). DMM surgery was performed to generate a mouse model for mimicking human OA. The articular cartilage degeneration was observed by safranin O-fast green staining at 8 weeks after operation. The OARSI scores of DMM-OA were apparently higher than that in sham group (*P* < 0.01, Fig. 2B). Real time PCR proved that CHMP5 expression was declined in the OA mouse articular cartilages at 4 and 8 weeks after operation compared with sham groups (*P* < 0.001, Fig. 2C). The results were also proved by IHC (*P* < 0.01, Fig. 2D). These findings illustrated that CHMP5 expression was descended in the articular cartilages of OA patients and mice.

### CHMP5 inhibits pro-apoptosis induced by IL-1β in chondrocytes

**Fig. 3:**
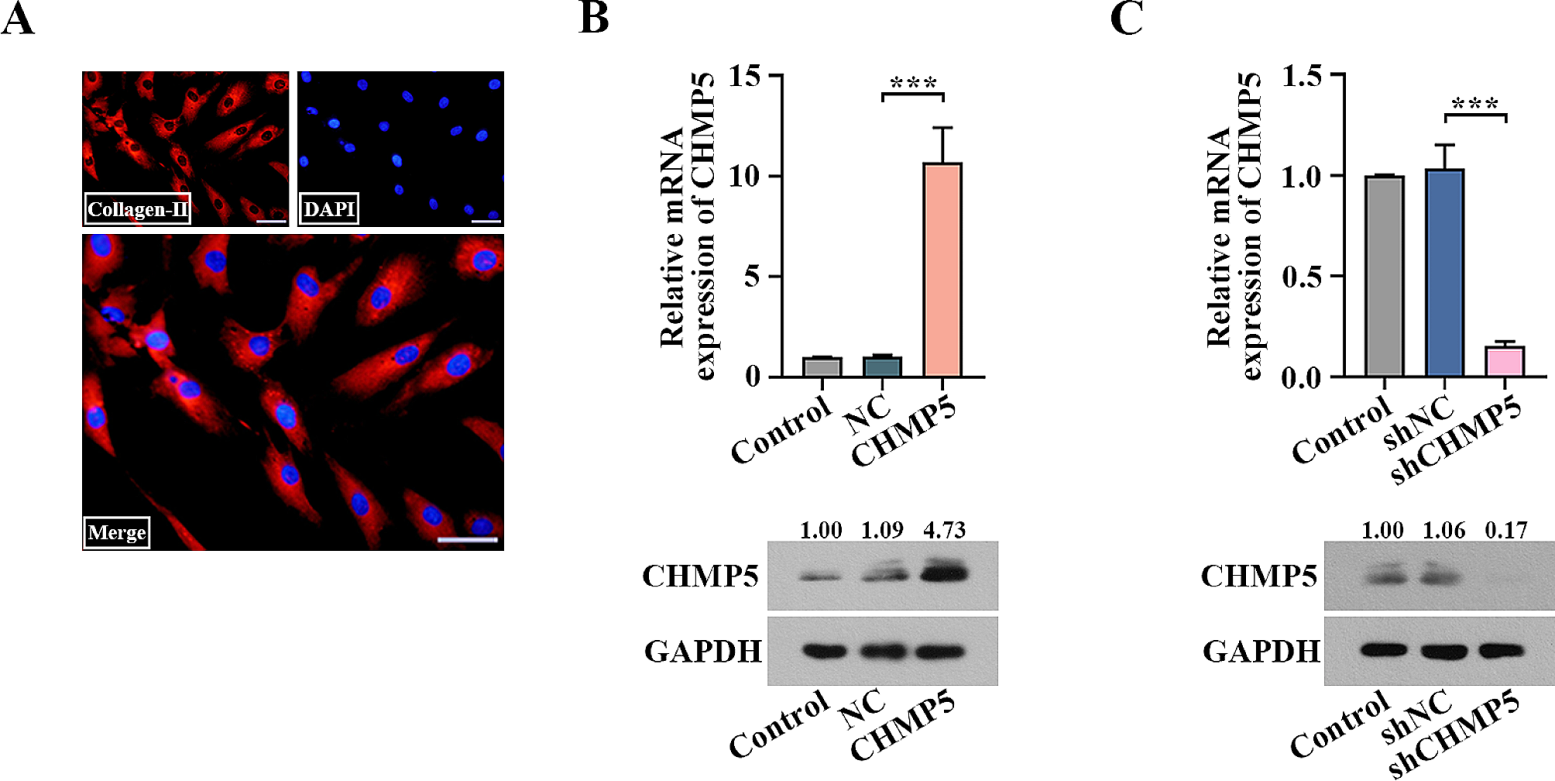
The identification of human chondrocytes and verification of transfection efficiency. (**A**) The chondrocytes were identified by detecting the levels of collagen-II using immunofluorescence (IF) assay. Bar: 50 μm. (**B**) The infection efficiency was detected by real-time PCR and western blot 24 h after the adenovirus infection. Adenovirus-mediated CHMP5 overexpression (Ad-CHMP5) increased the levels of CHMP5, and (**C**) CHMP5 small hairpin RNA (Ad-shCHMP5) decreased the levels of CHMP5 in the chondrocytes. ***P < 0.001

**Fig. 4:**
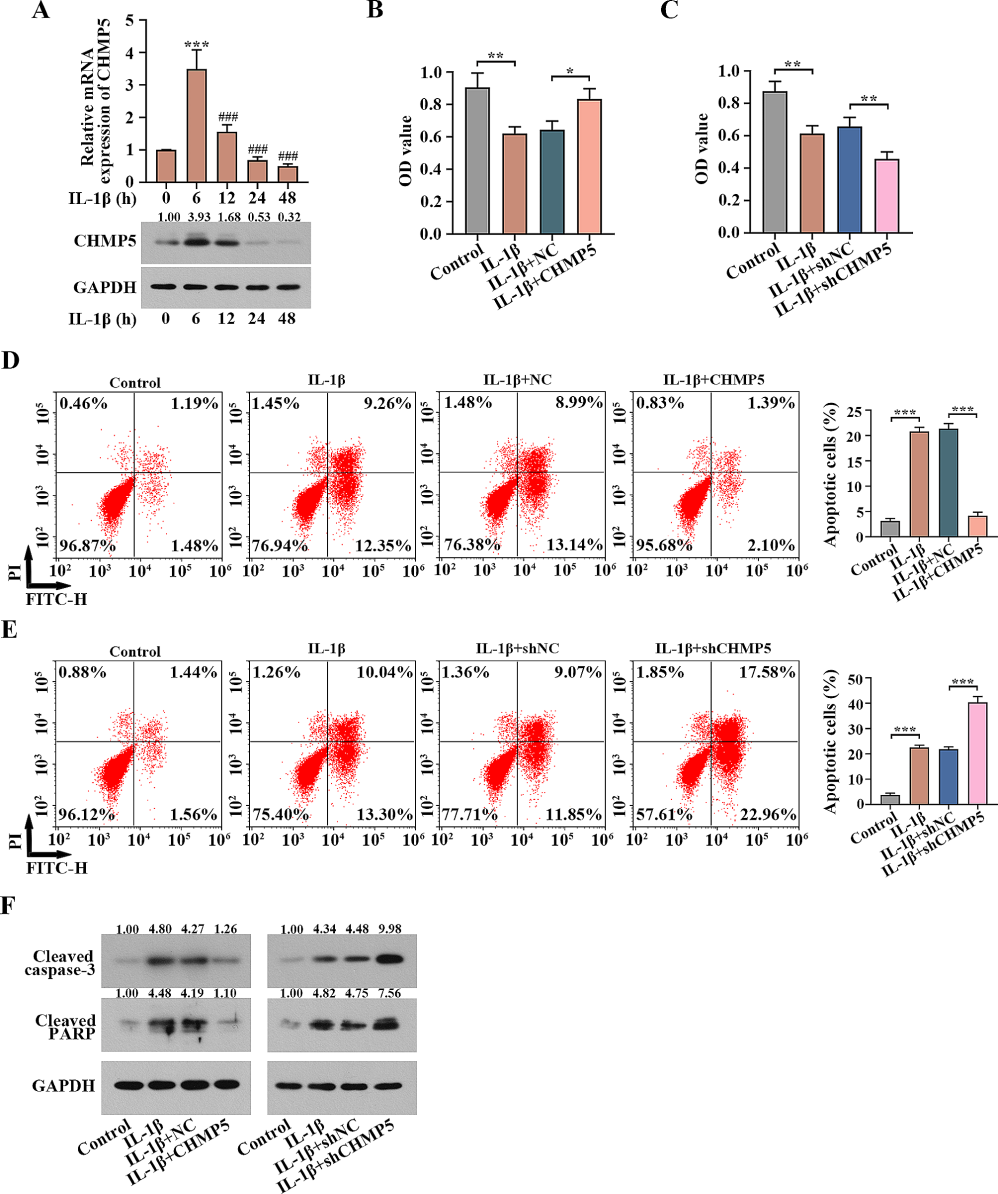
CHMP5 suppressed IL-1β-stimulated pro-apoptosis in the chondrocytes. (**A**) Expression of CHMP5 in the chondrocytes treated with IL-1β for different times was tested by real time PCR and western blot. ***P < 0.001 versus the 0 h group. ^###^*P* < 0.001 versus the 6 h group. (**B**) CHMP5 overexpression restored but (**C**) CHMP5 knockdown exacerbated the repression of chondrocyte viability induced by IL-1β. (**D**) CHMP5 overexpression reversed IL-1β-activated pro-apoptotic effect. (**E**) CHMP5 silencing promoted the effect. (**F**) The expression of cleaved caspase-3 and cleaved PARP were detected by western blot. *P < 0.05. **P < 0.01. ***P < 0.001

The chondrocytes were identified by detecting collagen-II levels using IF (Fig. 3A). Real time PCR and western blot showed the adenoviruses were infected into chondrocytes to induce CHMP5 overexpression and knockdown (*P* < 0.001, Fig. 3B and C). The mRNA and protein levels of CHMP5 were measured with IL-1β addition at 0 h, 6 h, 12 h, 24 h and 48 h. CHMP5 expression appeared to increase sharply at 6 h, and then it was reduced with the increase in IL-1β stimulation time. Notably, the levels of CHMP5 were inhibited significantly at 24 h in chondrocytes, which were used for the following detection (*P* < 0.001, Fig. 4A). The chondrocyte viability was repressed due to IL-1β stimulation, which was reversed by CHMP5 overexpression but was exacerbated by CHMP5 knockdown (*P* < 0.05, Fig. 4B and C). Flow cytometry analysis showed that overexpressing CHMP5 restrained the IL-1β-activated pro-apoptotic effect. By contrast, CHMP5 silencing boosted pro-apoptosis (*P* < 0.05, Fig. 4D and F). Pro-apoptotic markers, including cleaved caspase-3 and cleaved PARP, were examined by western blot, showing similar results (Fig. 4F). Thus, CHMP5 prohibited pro-apoptosis caused by IL-1β in human chondrocytes.

### CHMP5 diminishes IL-1β-triggered ECM degradation in chondrocytes

**Fig. 5:**
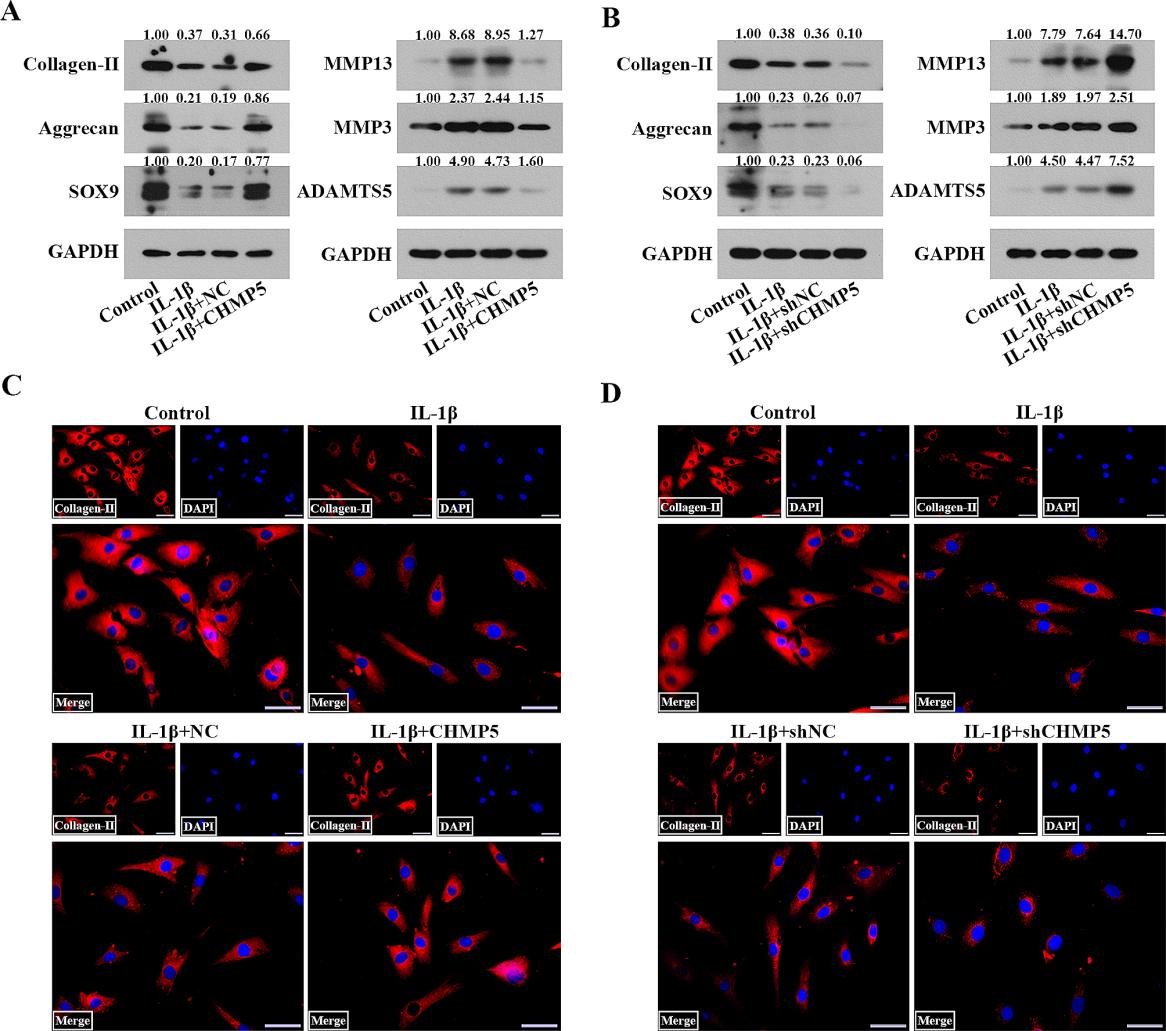
CHMP5 constricted ECM degradation induced by IL-1β in the chondrocytes. (**A, B**) Western blot showed the levels of collagen II, aggrecan, SOX9, MMP13, MMP3 and ADAMTS5 in the chondrocytes. (**C, D**) IF staining of collagen II exhibited the protective effect of CHMP5 on ECM in the chondrocytes. Bar: 50 μm

The reduction of matrix-synthesizing enzymes (collagen II, aggrecan and SOX9) and the increase of matrix-degrading enzymes (MMP13, MMP3 and ADAMTS5) induced by IL-1β were blocked in the chondrocytes transfected with Ad-CHMP5. The contrary tendency was exhibited when CHMP5 was silenced (Fig. 5A and B). As expected, IF staining showed that CHMP5 overexpression rescued IL-1β-stimulated collagen II degradation in chondrocytes (Fig. 5C and D). These results indicated that CHMP5 might protect chondrocytes against IL-1β-induced ECM catabolism.

### CHMP5 prevents IL-1β-induced NF-κB pathway activation in chondrocytes

**Fig. 6:**
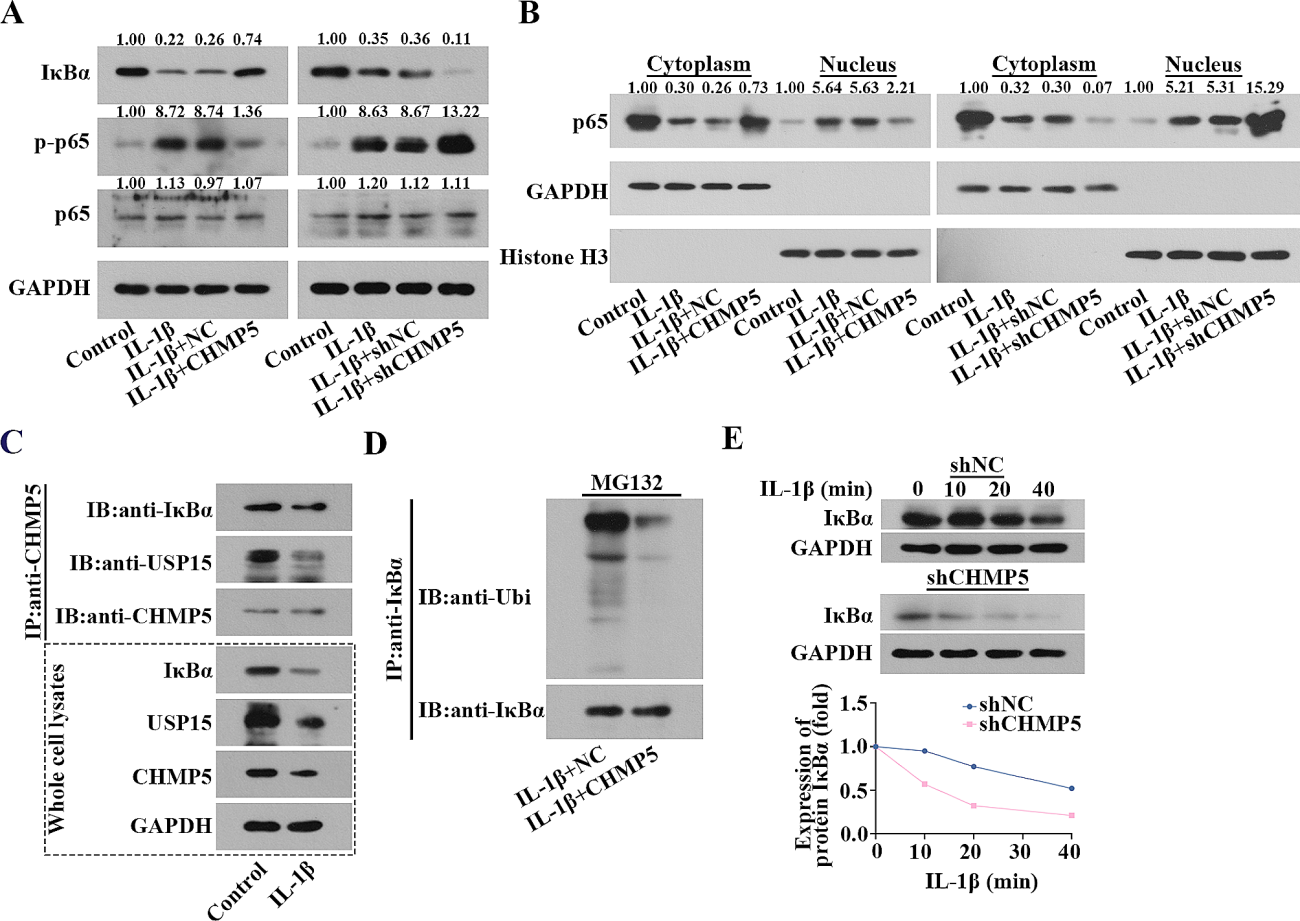
IL-1β-activated NF-κB pathway was restrained by CHMP5 in the chondrocytes. (**A**) The protein contents of IκBα, p-p65 and p65 were quantified in the chondrocytes by western blot. (**B**) CHMP5 reversed IL-1β-stimulated increase of p65 expression in the nucleus and decrease of p65 expression in the cytoplasm. (**C**) CHMP5 bound with IκBα and USP15 proteins in the chondrocytes. (**D**) CHMP5 decreased the ubiquitination of IκBα in the chondrocytes. (**E**) Silencing CHMP5 downregulated IκBα levels in the chondrocytes with the addition of IL-1β

To investigate whether CHMP5 regulates the IL-1β-induced NF-κB pathway in chondrocytes, we tested the contents of IκBα, p-p65 and p65 by western blot. The results manifested that CHMP5 recovered IL-1β-stimulated changes of IκBα and p-p65 in chondrocytes (Fig. 6A). In addition, up-regulating CHMP5 enhanced IL-1β-induced diminution of p65 in the cytoplasm and constricted increase of p65 in the nucleus while downregulating CHMP5 levels resulted in the opposite effects (Fig. 6B). CHMP5 was proved to be able to bind with IκBα and USP15 proteins through Co-IP assay in the chondrocytes with IL-1β (Fig. 6C). Moreover, CHMP5 overexpression decreased the ubiquitination of IκBα in the IL-1β-added chondrocytes co-treated with the proteasome inhibitor MG132 (Fig. 6D). Besides, silencing CHMP5 dropped IκBα levels in the chondrocytes with the treatment of IL-1β (Fig. 6E), suggesting that CHMP5 dampened the NF-κB pathway in the chondrocytes by binding USP15 and deubiquitinating IκBα.

### CHMP5 ameliorates OA development in DMM-mediated mice

**Fig. 7:**
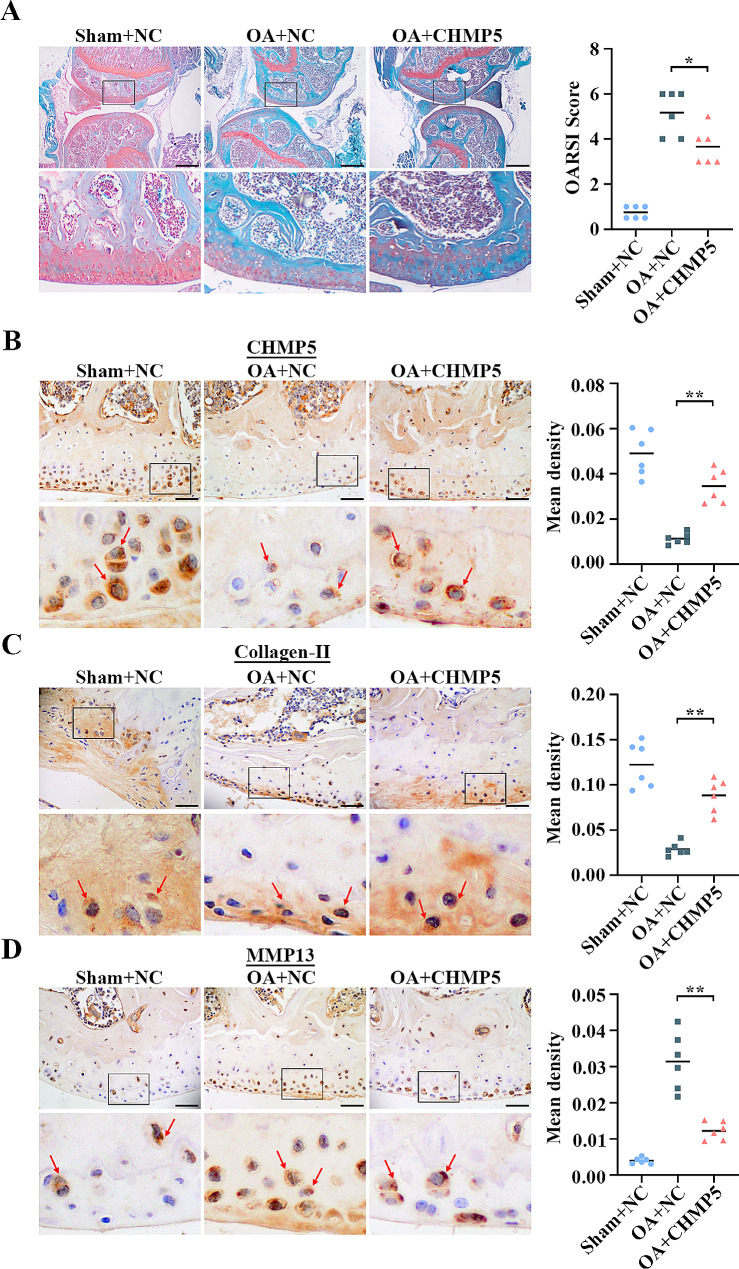
CHMP5 mitigated OA progression in the DMM-mediated mice. (**A**) CHMP5 diminished mouse cartilage destruction (Mann-Whitney test). Bar: 500 μm. (**B-D**) CHMP5, Collagen II, and MMP-13 were examined by IHC assay (Mann-Whitney test). Bar: 50 μm. *P < 0.05. **P < 0.01

To validate the protective effect of CHMP5 against OA, a DMM surgery-induced OA mouse model was established. Safranin O-fast green staining displayed that CHMP5 mitigated the knee joint destruction in OA mice (*P* < 0.05, Fig. 7A). According to the IHC results, overexpressing CHMP5 induced the increase of collagen II as well as the decrease of MMP-13 in the mouse cartilages (*P* < 0.01, Fig. 7B-D). These results further revealed that CHMP5 attenuated OA mouse cartilage destruction by inhibiting ECM degradation.

## Discussion

**Fig. 8:**
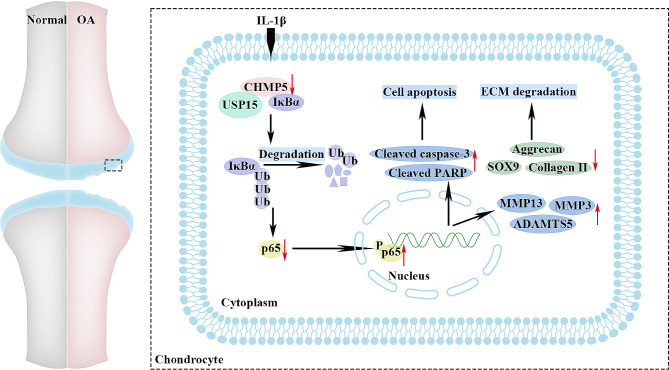
A schematic diagram showing that CHMP5 attenuates OA via inhibiting IL-1β-induced chondrocyte apoptosis and ECM degradation, involving NF-κB pathway

OA is an aging-associated degenerative disease, leading to disability globally (Hunter and Bierma-Zeinstra 2019; Palazzo et al. 2016). Identifying new molecular targets is increasingly vital to the diagnosis and therapy of OA. In our study, we identified a downregulated CHMP5 in the articular cartilages of OA patients by proteomic analysis, and it was verified in human and mouse cartilages. The chondrocytes were exposed to IL-1β to result in apoptosis and ECM degradation, which was rescued by adenovirus-mediated CHMP5 overexpression. However, silencing CHMP5 aggravated the adverse impacts. Moreover, we found that the protection of CHMP5 against OA was involved in NF-κB pathway (Fig. 8). Further experiments in DMM-operated OA mice exhibited the same results. Our data implicated that CHMP5 might serve as a novel therapeutic target for OA.

Chondrocytes are the only cells distributed in the ECM in cartilage. The survival of chondrocytes is vital for ECM homeostasis (Hwang and Kim 2015). Heraud et al. have found that 18 − 21% of chondrocytes show apoptotic features in OA cartilage compared with 2 − 5% in normal cartilage (Héraud et al. 2000). It is indicated that apoptosis may be a new target for treatment of OA. As well as apoptosis, impaired synthesis and catabolism of ECM also lead to OA. The synthesis of ECM is partially mediated by a variety of matrix-synthesizing enzymes, including collagen II, aggrecan and SOX9 (Lefebvre and de Crombrugghe 1998). In addition, MMP13, MMP3 and ADAMTS5 are the primary mediators of ECM catabolism (Glasson et al. 2005; Hao et al. 2022). The degradation of the ECM and the apoptosis of chondrocytes are two crucial pathogenic events in OA.

According to proteomics analysis, we identified 86 up-regulated and 65 down-regulated differentially expressed proteins in the OA group compared with the control group. Especially several proteins, such as C-C motif chemokine ligand (Subburaman and Edderkaoui 2021), G protein-coupled receptor 4 (Li et al. 2022), aggrecan (Dunn et al. 2016), apolipoprotein A1 (de Seny et al. 2015), collagen type 3 alpha 1 chain (Li et al. 2021), orosomucoid 2 (Blanco et al. 2019), and myristoylated alanine rich protein kinase C substrate (Chen et al. 2018), have been reported in OA. We focused on researching CHMP5, which has not been reported in OA. CHMP5 is a component of the endosomal sorting complex required for the transport III (ESCRT-III) complex, which is essential for the repair of damaged plasma membranes in various types of regulated cell death, such as necroptosis and ferroptosis (Liu et al. 2021; Yang et al. 2022). There are very few research reports on the involvement of CHMP5 in disease progression. For example, CHMP5 serves as an anti-apoptotic protein in leukemogenesis. The Granzyme B/Perforin apoptotic pathway is activated in CHMP5-deficient leukemic cells (Wang et al. 2013). Additionally, Shahmoradgoli et al. propose that CHMP5 may be an oncogenic gene in acute myeloid leukemia due to the anti-apoptotic feature of CHMP5 by inhibiting apoptosis-associated genes and different signaling pathways, including NF-κB (Shahmoradgoli et al. 2011). These studies suggest that CHMP5 may be closely related to cell apoptosis and NF-κB signaling pathways. In the study, we found overexpressed CHMP5 reduced an increase in cleaved caspase 3 induced by IL-1β in chondrocytes. However, knocking down CHMP5 exhibited an inverse trend. In addition, CHMP5 prevented IL-1β-induced NF-κB pathway activation in chondrocytes. Thus, it was demonstrated that the inhibitory effect of CHMP5 on OA progression also involves apoptosis and NF-κB pathway.

In recent decades, numerous studies have identified several signaling pathways involving OA pathophysiology, such as Wnt/β-catenin signaling pathway (Miyatake et al. 2020; Wang et al. 2019), PI3K/Akt/mTOR signaling pathway (Xu et al. 2021) and NF-κB signaling pathway (Chang et al. 2019; Choi et al. 2019; Guo et al. 2021). Among them, NF-κB signaling is a widely studied pathway participating in the IL-1β-induced OA model (Choi et al. 2019; Jimi et al. 2019). NF-κB signaling is activated in OA chondrocytes during aging and inflammation (Marcu et al. 2010). The cyclic GMP-AMP synthase-stimulator of interferon genes facilitates chondrocyte senescence, apoptosis, and ECM degradation in OA, involving the NF-κB signaling pathway (Guo et al. 2021). Intriguingly, NF-κB is an essential signaling pathway that responds to chondrocyte inflammatory cytokines, such as IL-1β (Choi et al. 2019; Lepetsos et al. 2019). Insulin-like growth factor and platelet-derived growth factor suppress IL-1β-activated cartilage degradation via down-regulation of NF-κB signaling pathway, which causes downstream effects such as apoptosis and ECM metabolism imbalance (Montaseri et al. 2011). CHMP5 requires the deubiquitinating enzyme USP15 to stabilize IκBα, which constricts NF-κB activation and thus represses osteoclast differentiation, osteoblast coupling and bone turnover rates (Greenblatt et al. 2015). Therefore, we speculated whether CHMP5 regulates OA progression by binding to USP15 and NF-κB signaling pathway. As expected, our current investigation showed that CHMP5 was capable of binding USP15 to inhibit IκBα ubiquitination, thereby suppressing enhanced apoptosis and ECM degradation induced by IL-1β through NF-κB signaling pathway in OA chondrocytes.

Taken together, CHMP5 alleviates OA development by decreasing OA chondrocyte apoptosis and ECM degradation caused by IL-1β via NF-κB signaling pathway (Fig. 8). It was indicated that CHMP5 might be a potential therapeutic target for OA.

### Electronic supplementary material

Below is the link to the electronic supplementary material.

**Additional file 1**: **Fig. S1**A. Quality control of control (C1-3) and OA (OA1-3) samples for TMTTM quantitative proteomics analysis. (A) A matrix of scatter plots and Pearson correlation coefficient of protein intensities for each sample. (B) Histogram of log2 protein intensity for each sample in TMTTM-based proteomic analysis. (C) A heat map in control and OA groups. Red represented the high expression, and blue represented the low expression.

## Data Availability

The datasets used and/or analyzed during the current study are available from the corresponding author upon reasonable request.

## Abbreviations

CHMP5: Charged multivesicular body protein 5
NF-κB: Nuclear factor kappa-B
OA: Osteoarthritis
ECM: Extracellular matrix
DMM: Destabilization of the medial meniscus
IL-1β: Interleukin-1 beta
ADAMTS: A disintegrin and metalloprotease with thrombospondin motifs
MMPs: Matrix metalloproteinases
PNAS-2: Peptide nucleic acids 2
CHMP: Chromatin-modifying protein/charged multivesicular body protein
TNFα: Tumor necrosis factor alpha
FBS: Fetal bovine serum
DMEM/F12: Dulbecco’s Modified Eagle Medium/Nutrient Mixture F-12
OARSI: Osteoarthritis Research Society International
IHC: Immunohistochemistry
MMP13: Matrix metallopeptidase 13
HRP: Horseradish peroxidase
GAPDHs: Glyceraldehyde 3-phosphate dehydrogenases
CCK-8: Cell counting kit 8
PE: Phycoerythrin
RIPA: Radioimmunoprecipitation assay
PMSF: Phenylmethanesulfonyl fluoride
SDS-PAGE: Sodium dodecyl sulphate polyacrylamide gel electrophoresis
PVDF: Polyvinylidene difluoride
PARP: Poly (ADP-ribose) polymerases
MMP3: Matrix metallopeptidase 3
PCA: Principal component analysis
KEGG: Kyoto Encyclopedia of Genes and Genomes
GO: Gene Ontology
ESCRT-III: Endosomal sorting complex required for transport III
