# Supplementary figures and images for: CHMP5 attenuates osteoarthritis via inhibiting chondrocyte apoptosis and extracellular matrix degradation: involvement of NF-κB pathway
Source: Mol Med. 2024 Apr 25;30:55. doi: 10.1186/s10020-024-00819-6 (PMC11046779; doi:10.1186/s10020-024-00819-6)

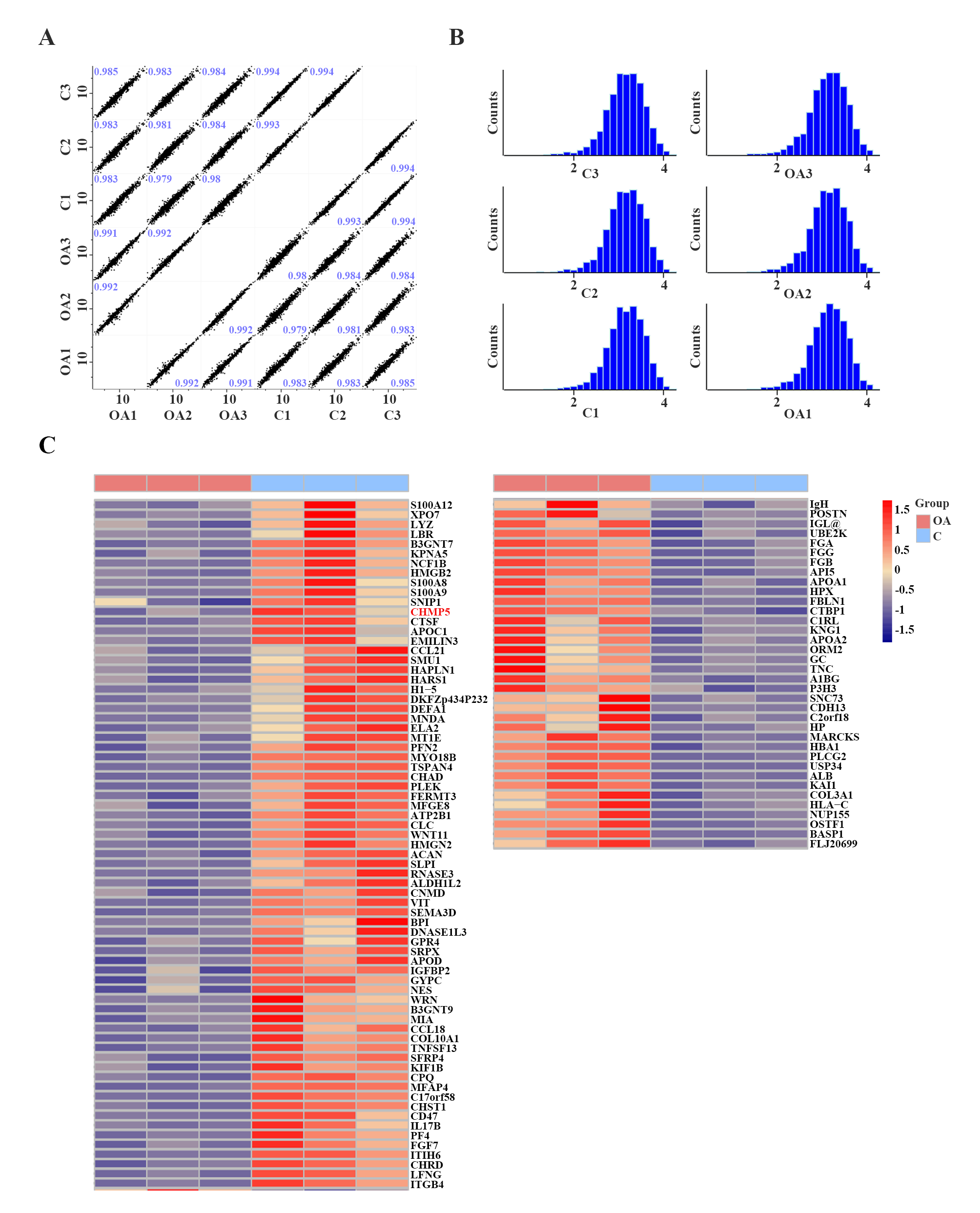

Supplement: Supplementary file 1 — Additional file 1: Fig. S1A. Quality control of control (C1-3) and OA (OA1-3) samples for TMTTM quantitative proteomics analysis. (A) A matrix of scatter plots and Pearson correlation coefficient of protein intensities for each sample. (B) Histogram of log2 protein intensity for each sample in TMTTM-based proteomic analysis. (C) A heat map in control and OA groups. Red represented the high expression, and blue represented the low expression. [file 10020_2024_819_MOESM1_ESM.tif]
